# A spatial analysis of TB cases and abnormal X-rays detected through active case-finding in Karachi, Pakistan

**DOI:** 10.1038/s41598-023-28529-9

**Published:** 2023-01-24

**Authors:** Syed Mohammad Asad Zaidi, Wafa Zehra Jamal, Christina Mergenthaler, Kiran Sohail Azeemi, Nick Van Den Berge, Jacob Creswell, Aamir Khan, Saira Khowaja, Shifa Salman Habib

**Affiliations:** 1 Community Health Solutions, Karachi, Pakistan; 2 KIT Royal Tropical Institute, Amsterdam, The Netherlands; 3 The Stop TB Partnership, Geneva, Switzerland; 4 IRD Global, Singapore, Singapore; 5 Indus Health Network, Karachi, Pakistan

**Keywords:** Population screening, Tuberculosis

## Abstract

Tuberculosis (TB) is the leading cause of avoidable deaths from an infectious disease globally and a large of number of people who develop TB each year remain undiagnosed. Active case-finding has been recommended by the World Health Organization to bridge the case-detection gap for TB in high burden countries. However, concerns remain regarding their yield and cost-effectiveness. Data from mobile chest X-ray (CXR) supported active case-finding community camps conducted in Karachi, Pakistan from July 2018 to March 2020 was retrospectively analyzed. Frequency analysis was carried out at the camp-level and outcomes of interest for the spatial analyses were mycobacterium TB positivity (MTB+) and X-ray abnormality rates. The Global Moran’s I statistic was used to test for spatial autocorrelation for MTB+ and abnormal X-rays within Union Councils (UCs) in Karachi. A total of 1161 (78.1%) camps yielded no MTB+ cases, 246 (16.5%) camps yielded 1 MTB+, 52 (3.5%) camps yielded 2 MTB+ and 27 (1.8%) yielded 3 or more MTB+. A total of 79 (5.3%) camps accounted for 193 (44.0%) of MTB+ cases detected. Statistically significant clustering for MTB positivity (Global Moran’s I: 0.09) and abnormal chest X-rays (Global Moran’s I: 0.36) rates was identified within UCs in Karachi. Clustering of UCs with high MTB positivity were identified in Karachi West district. Statistically significant spatial variation was identified in yield of bacteriologically positive TB cases and in abnormal CXR through active case-finding in Karachi. Cost-effectiveness of active case-finding programs can be improved by identifying and focusing interventions in hotspots and avoiding locations with no known TB cases reported through routine surveillance.

## Introduction

Tuberculosis (TB) is the leading cause of avoidable deaths from an infectious disease globally. In 2019, an estimated 10 million people developed TB and 1.5 million died from the disease^1^. A main reason for the high mortality is that a large of number of people who develop TB each year remain undiagnosed. For several decades, TB control was based on a passive approach where existing healthcare facilities diagnosed and treated people presenting with symptoms of TB. This strategy had several limitations as people often did not seek care due to poor knowledge of the disease, stigma or health access barriers. In addition, when people did seek care, they often presented at an advanced stage of the disease leading to poorer treatment outcomes and increased community transmission^2^. Recently, active case-finding (ACF) has been recommended by the World Health Organization (WHO) to bridge the case-detection gap for TB in high burden countries^3^. This approach involves screening for TB among people who may not actively seek healthcare, through additional services, often outside of health facilities.

Pakistan has the world’s fifth highest TB burden and only 58% of the people estimated to have TB were detected and notified in 2019^4^. The Government of Pakistan aims to eliminate TB by 2035 and increasing case-detection is a key objective. In recent years, the National TB Program (NTP) that oversees TB elimination efforts in the country, has prioritized ACF to increase the number of cases diagnosed and reduce community transmission of TB. The capacity for ACF has been significantly strengthened in Pakistan through capital investments for mobile X-rays vans by NTP and its private-sector implementation partners^5^. However, concerns remain regarding their yields and cost-effectiveness^6^. As with any health-related intervention, TB programs in high burden countries such as Pakistan must rationalize costs given many competing priorities, and therefore tools and approaches to improve the efficiency of ACF interventions are particularly useful^2^.

The use of geographic information systems (GIS) with spatial statistics has been applied to analyze spatial patterns of several infectious diseases^7–9^. While TB incidence and prevalence estimates are calculated at national or regional levels, TB epidemics may be characterized by patches of concentrated risk at the local level rather than spatially uniform risk. Investigating spatial heterogeneity in TB incidence and concentrating interventions in locations of high risk may improve the cost-effectiveness of ACF^10,11^. Modelling studies suggest that targeting ACF only in TB hotspots, has a similar impact on reducing TB incidence as conducting TB control interventions over an entire city^12–14^. However, in practice, identifying TB hotspots is challenging in low and middle income countries, given the absence of street addresses and electronic registries for TB patients. In addition, data sources often largely reliant on passive case-detection registers that may not accurately reflect areas of high disease transmission by excluding asymptomatic carriers in early or subclinical phases of TB^2,15,16^.

In 2017, an ACF program was initiated in Karachi, Pakistan supported by mobile vans equipped with digital chest X-rays and diagnostic testing using Xpert MTB/RIF (Xpert)^17^. This program provided an opportunity to evaluate spatial variation in TB risk during a large-scale ACF intervention from a South-Asian megacity and to identify potential hotspots. In this study, we describe spatial dependence of bacteriologically positive TB (primary outcome) and abnormal X-ray cases (secondary outcome) detected during the ACF program. In the absence of localized prevalence surveys that can be very costly to implement, the outcomes selected in this study served as proxy measures of neighborhood-level TB prevalence and risk in Karachi.

## Results

**Figure 1:**
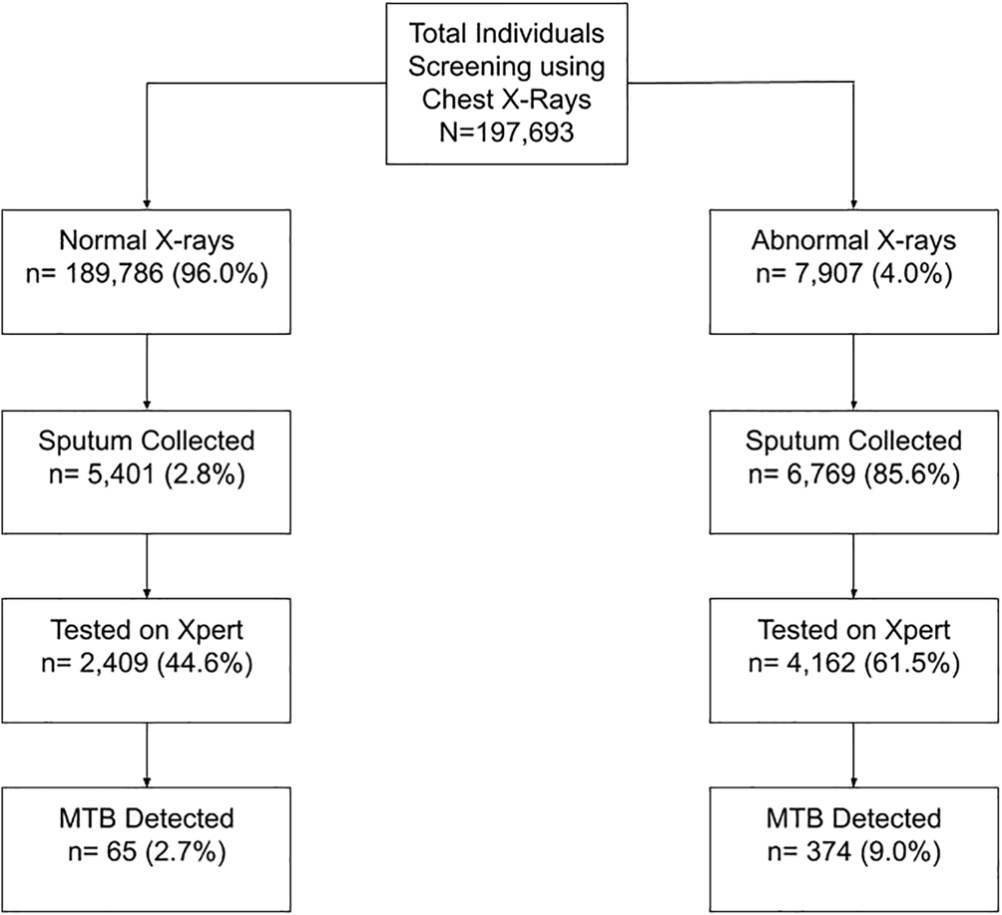
Overview of TB case-detection cascade for mobile chest X-ray supported active case-finding camps in Karachi, Pakistan (July 2018–March 2020).

A total of 1543 chest camps, in 151 union councils, were conducted during the study period and included in the analysis. A total of 57 (3.7%) camps did not have GPS coordinates and were excluded from the analysis. A total of 197,693 individuals were screened using CXR among whom 7907 (4%) were deemed abnormal (Fig. 1). The median age of camp participants was 35 (IQR 13.8) and the age distribution was skewed towards older age groups (Supplementary Fig. 1). Among those screened 78.3% were males (Table 1).

**Table 1:** Participant demographics and TB case-detection cascade indicators for mobile chest X-ray supported active case-finding camps in Karachi, Pakistan (July 2018–March 2020).

	n/mean/median	IQR/SD/%
Age of participants	35	13.8
Proportion males	78.3%	–
Participants per camp	139	(98–175)
Abnormal X-rays per camp	5	(2–7)
Sputum samples collected per camp	6	(3–11)
Xpert MTB/RIF tests per camp	4	(2–6)
MTB + detected per camp	0.29	0.65

The median number of participants per chest camp was 139 (IQR 98–175). Sputum was collected from 6769 (85.6% of individuals with an abnormal X-ray) and from an additional 5401 people (2.8% of those with a normal X-ray). After evaluating sputum quality, a total of 4162 Xpert tests were conducted among individuals with an abnormal X-ray yielding 374 MTB+ cases (9% positivity), whereas 2409 Xpert tests were conducted among individuals with a normal X-ray yielding, 65 MTB+ cases (2.7% positivity). A median of 5 (IQR 2–7) abnormal chest X-rays and 6 (IQR 3–11) sputum samples were collected per chest camp. Each camp yielded a mean of 0.29 (± 0.65) individuals with MTB+ results. A total of 1161 (78.1%) camps yielded no MTB+ cases, 246 (16.5%) camps yielded 1 MTB+, 52 (3.5%) camps yielded 2 MTB+ and 27 (1.8%) yielded 3 or more MTB+ (Table 2). A total of 79 (5.3%) camps accounted for 193 (44.0%) of MTB+ cases detected.

**Table 2:** Yield of TB case-detection from mobile chest X-ray supported active case-finding camps in Karachi, Pakistan (July 2018–March 2020).

	n	%	MTB + detected	%
Camps with zero MTB+	1161	78.1	0	0
Camps with 1 MTB+	246	16.5	246	61.3
Camps with 2 MTB+	52	3.5	104	23.7
Camps with 3 or more MTB+	27	1.8	89	20.3
Total camps	1486		439	

**Figure 2:**
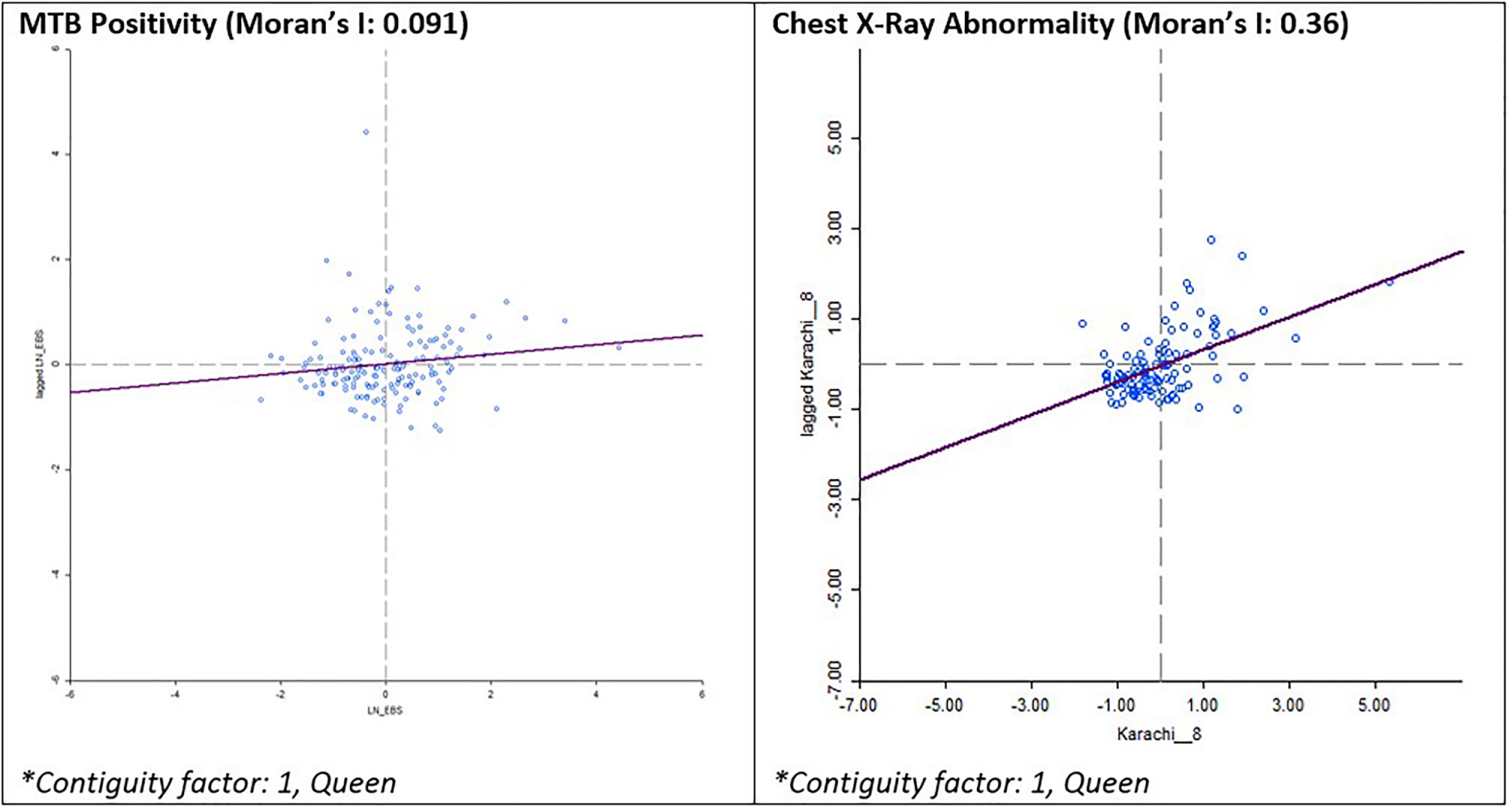
Global Moran’s I statistic for MTB positivity and X-ray abnormality rates from mobile chest X-ray supported active case-finding camps in Karachi, Pakistan (July 2018–March 2020).

**Figure 3:**
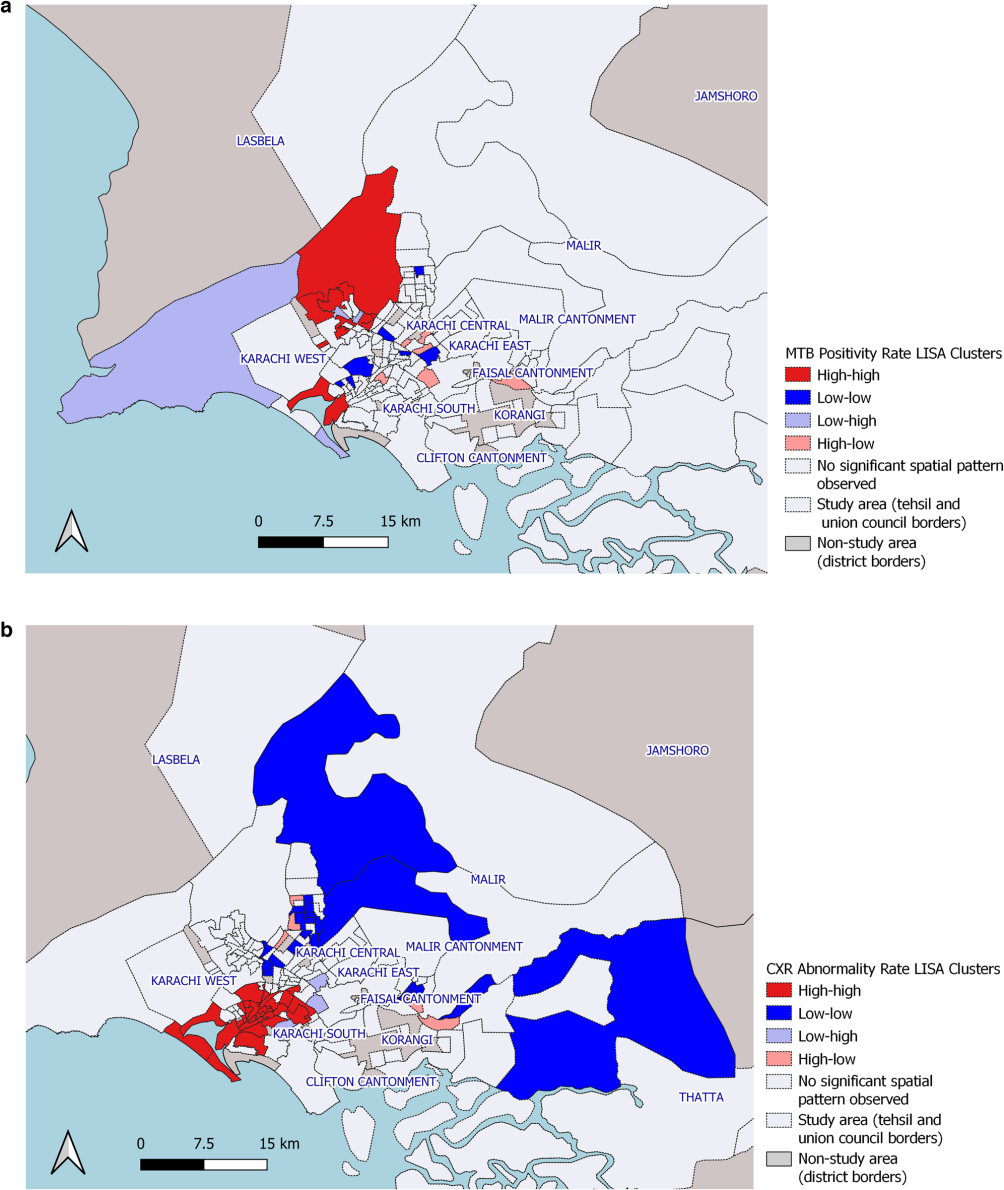
(**a**) Local Indicators of Spatial Association (LISA) analysis for MTB positivity rates in Union Councils (UCs) from mobile chest X-ray supported active case-finding camps in Karachi, Pakistan (July 2018–March 2020). A High-high result indicates potential clustering of UCs with high MTB positivity. A High-low result indicates a UC with a potentially high MTB positivity surrounded by UCs with low positivity. A low–low result indicates clustering of UCs with potentially low MTB positivity. A low–high result indicates a UC with potentially low MTB positivity surrounded by UCs with high positivity. QGIS Geographic Information System v3.26.3. QGIS.org, 2022. QGIS Association. http://www.qgis.org. (**b**) Local Indicators of Spatial Association (LISA) analysis for abnormal X-ray rates in Union Councils (UCs) from mobile chest X-ray supported active case-finding camps in Karachi, Pakistan (July 2018–March 2020). A High-high result indicates clustering of potential UCs with high abnormal X-ray rates. A High-low result indicates a UC with potentially high abnormal X-ray ratio surrounded by UCs with low rates. A low–low result indicates clustering of UCs with low abnormal X-ray rates. A low–high result indicates a UC with potentially low MTB positivity surrounded by UCs with high positivity. QGIS Geographic Information System v3.26.3. QGIS.org, 2022. QGIS Association. http://www.qgis.org.

The local Moran’s I statistic identified statistically significant clustering for MTB positivity rates within UCs in Karachi overall (local Moran’s I: 0.092; z-score = 1.9957; pseudo p-value = 0.036) and statistically significant clustering for abnormal chest X-ray rates (local Moran’s I: 0.363; z-score = 7.3569; pseudo p-value = 0.001) (Fig. 2). The LISA analysis identified clustering of UCs with high MTB+ rates (described as High-high) in Karachi West and Karachi South using a contiguity of 1 (Fig. 3a). Union Councils with high MTB+ surrounded by UCs with low rates (described as High-Low) were identified in Karachi East, Central and Korangi. These suggest locations with potential hotspots where screening can be targeted (Table 3). Clustering of UCs with high abnormal chest x-ray rates were identified in Karachi West and Karachi South (Table 4). Clustering of UCs with low MTB+ and abnormal X-ray rates (described as low–low) were identified primarily in Karachi Central district (Fig. 3b).

**Table 3:** Potential locations with clustering of MTB positivity identified from mobile chest X-ray supported active case-finding camps in Karachi, Pakistan (July 2018–March 2020).

District	Tehsil	Locations with clustering of MTB+ cases	Type
Malir	Gadap Town	Mangho Pir	High–high
Karachi West	Baldia Town	Ittehad Town, Mohajir Camp	
	Orangi Town	Chushti Nagar, Ghaziabad, Baloch Goth, Mujahidabad, Haryana Colony, Muhammad Nagar, Dad Nagar, Bilal Colony	
	Kemari Town	Baba Bhitt	
Karachi South	Jamshed Town	Soldier Bazar	High–low
Karachi East	Gulshan E Iqbal Town	Dehli Mercantile, Jamali Colony	
Korangi	Shah Faisal Town	Al Falah Society	
Karachi Central	Gulberg Town	Yaseenabad, Karimabad	
	Liaqatabad Town	Abbasi Shaheed

**Table 4:** Potential locations with clustering of abnormal X-rays identified from mobile chest X-ray supported active case-finding camps in Karachi, Pakistan (July 2018–March 2020).

District	Tehsil	Locations with clustering of abnormal chest X-rays	Type
Karachi West	Kemari Town	Baba Bhitt, Bhutta Village, Sultanabad	High–high
	Site Town	Islamia Colony	
Karachi South	Saddar Town	Railway Colony, Kharadar, Garden	
	Lyari	Lyari, Khada Memon	
	Jamshed Town	Jacob Lines	
Karachi Central	North Nazimabad Town	Buffer Zone	High–low
Malir	Bin Qasim Town	Ghaghar Phatak	

**Figure 4:**
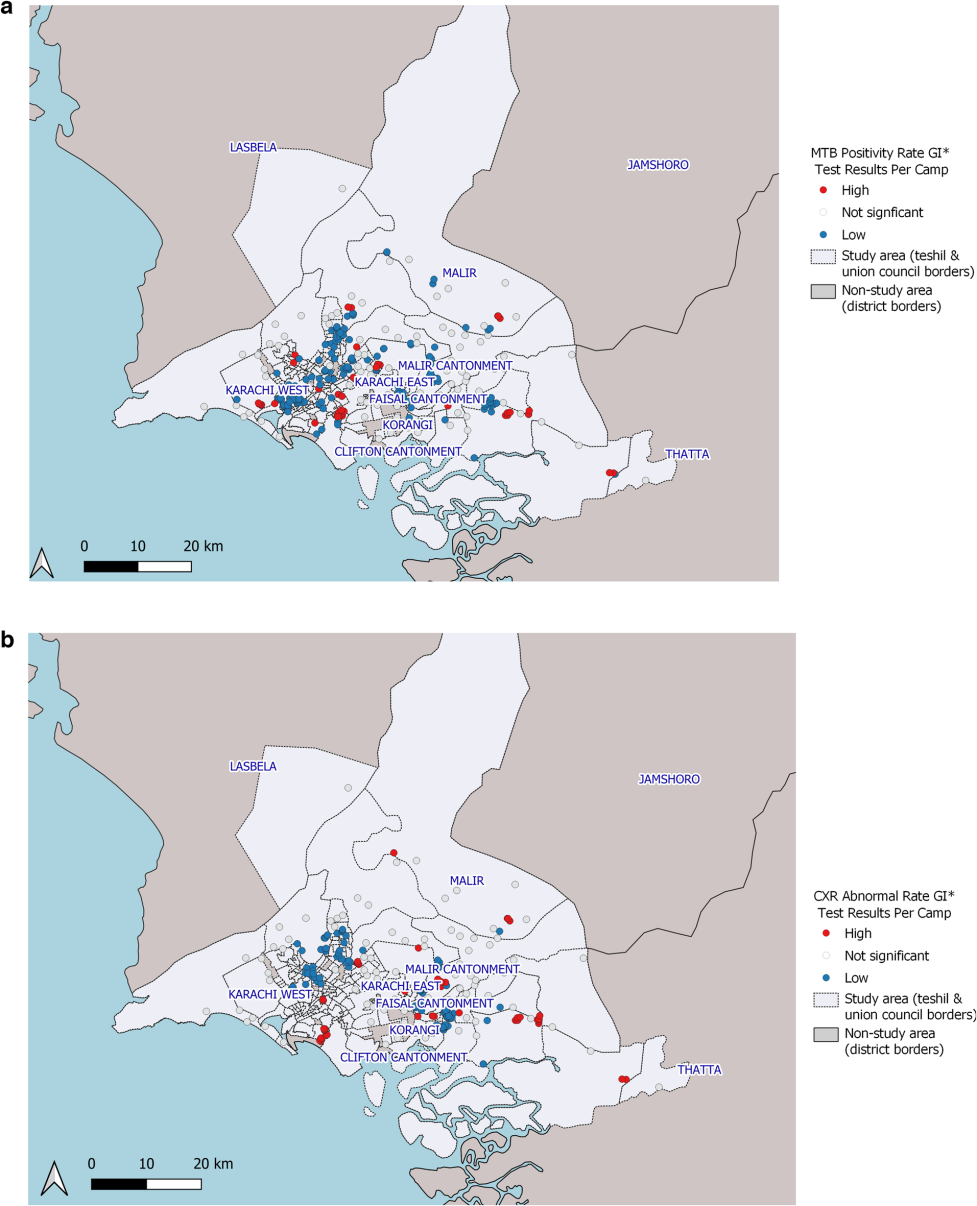
(**a**) GI* analysis (distance-based method) for MTB positivity rates from locations of mobile chest X-ray supported active case-finding camps in Karachi, Pakistan (July 2018–March 2020). A high–high result indicates clustering of GPS locations of camps with high MTB positivity. A low–low result indicates clustering of GPS locations of camps with low MTB positivity. QGIS Geographic Information System v3.26.3. QGIS.org, 2022. QGIS Association. http://www.qgis.org. (**b**) GI* analysis (distance-based method) for abnormal X-rays rates from locations of mobile chest X-ray supported active case-finding camps in Karachi, Pakistan (July 2018–March 2020). A high–high result indicates clustering of GPS locations of camps with high abnormal X-ray rates. A low–low result indicates clustering of GPS locations of camps with low abnormal X-ray rates. QGIS Geographic Information System v3.26.3. QGIS.org, 2022. QGIS Association. http://www.qgis.org.

The GI* analyses using both contiguity and distance-based weights identified clusters of camp locations with high MTB+ rates in Karachi South and Karachi West districts and in several locations in the north and eastern peripheries of the city that are part of District Malir (Supplementary Table 1). Clusters of camp locations with low MTB+ (described as low–low) were identified in District Central, District East and parts of Korangi district (Fig. 4a). Clusters of camp locations with high rates of abnormal X-rays identified in Karachi South and Malir districts (Supplementary Table 2). Clusters of camp locations with low abnormal X-ray rates were identified in Karachi West and Central districts (Fig. 4b). Abnormal X-rays were positively associated with MTB + using a logistic regression analysis (OR 3.30, 95% CI 1.75–6.20).

## Discussion

The premise of the Zero TB Cities Initiative is that concentrating resources in urban centers rather than spreading them over national or regional geographies can “drive sharp reductions in TB death rates and prevalence”^18^. This study provides evidence that even within cities, especially larger ones such as Karachi, the risk of TB is likely not distributed uniformly. While district-level GIS analyses have been previously carried out in Pakistan, this is the first study to investigate spatial variation in a major metropolitan area with detailed coordinates of ACF sites^19,20^. The strength of this study was the use of a very large dataset from an ACF program to achieve greater precision in the identification of TB hotspots in a city with diverse ethnicities and large variations in socioeconomic indicators. The ACF dataset allowed for inclusion of cases from the community that would otherwise been missed, thereby increasing the accuracy of our findings, relative to data captured from routine surveillance.

Our approach may allow for more actionable information that can be utilized to guide programmatic decision-making, further enhancing the Zero TB City approach. A notable finding was that greater than three-quarters of all camp locations yielded no MTB+ cases, whereas only 5% of camp locations accounted for over 40% of all MTB+ cases detected. These results strongly suggest a targeted approach to ACF in high-risk areas to improve yields and cost-effectiveness. A number of UCs with clustering of MTB yield were identified, particularly in the western and southern parts of the city. Clusters were also identified through the spatial point-pattern analysis using camp GPS coordinates in the western and southern regions, as well as in the peripheries of the city. These areas correspond to densely populated areas near the port, slum-dwellings in the west and peri-urban communities and villages in the outskirts of the city. It is likely that population density as well as social determinants of TB, such as crowded housing, low-income and poor nutrition contributed to higher TB risk^21,22^. UCs and camp locations with clustering of low values were identified in central and some eastern parts of the city. While this may suggest lower TB risk, a number of other factors may have also contributed towards lower yields. These locations were centered on major avenues of the city including commercial properties and planned middle and upper-middle income residential areas where residents have access to alternative healthcare facilities. Poor transportation links may have prevented people from reaching camp sites in other low value clusters. Fewer low–low clusters were identified for abnormal CXRs and this could be due to lower specificity of CXR relative to Xpert leading to false positives in areas that were cold-spots for TB.

Previous studies utilizing passive case-finding data from Brazil, South Africa and Zimbabwe have identified areas of high TB notifications in peri-urban and lower-income areas within cities^23–25^. A modelling study from Ho Chi Minh City found that four-fifths of index cases had no other reported TB cases within a 50 m radius^11^. These studies and our findings suggest that paying attention to potential TB hotspots may be a useful strategy for improving cost-effectiveness of ACF. However, notification rates from passive case-finding may not be a reliable measure of TB prevalence as estimates may be biased towards areas with higher health facility coverage^19^. Data from an ACF intervention therefore adds greater value in finding hotspots by limiting confounding caused by health systems and access factors.

Further research will be required to investigate causes for spatial heterogeneity within our study population. This can include social determinants such as population density, poverty, household family size and type of housing^26^. Investigating and addressing these factors would require a multi-disciplinary approach and collaboration with researchers involved in urban planning, housing and development as well as coordination with local city officials. Known clinical determinants of TB disease including nutritional deficiencies, smoking history, diabetes and HIV should also be examined for spatial heterogeneity^26–31^. Such analyses can be overlaid with this analysis and modeled as predictors for spatial variation in TB detection. Prospective studies should investigate ACF yields in areas considered to be at higher risk for TB through predictive modeling and improvements in cost-effectiveness from targeted screening should be analyzed.

Our approach can be easily replicated by other programs through the use of simple android mobile-phone applications and collection of GPS coordinates in the field. Free of cost tools such as Google Maps can be utilized to visualize color-coded clusters of camps yielding TB cases if propriety software is not available. Software code for mobile-applications utilized in this study is publicly available to support such data collection for field-teams in other settings. Revisions to the national active case-finding guidelines are also being prepared in collaboration with partners and the NTP to support the wider adoption of these methods. A similar analysis is being carried out for other cities in Pakistan where Community Health Solutions (CHS) operates.

There are a number of limitations in our analysis. The location of camp site was taken as a proxy for residence of the participants and this limits the internal validity of the study. While camps were carried out in communities and partnering provider clinics, it is possible that some participants were visiting the area and did not reside near the camp site or in the same UC. This may have resulted in a neighboring UC being incorrectly categorized as high or low risk. Most patients in our target population did not have accurate residential addresses and were not aware of their UCs, making it challenging to accurately map patient homes for such a large program. MTB and abnormal X-rays rates from the camps were approximated to the UC-level and may not accurately reflect the true underlying TB prevalence. A random sampling of households at the UC-level will provide a better estimate of TB prevalence, however, prevalence surveys require even more significant resources than an ACF intervention. Given the size of Karachi’s population and diversity of its neighborhoods, our results support investment in a city-level prevalence survey to help identify areas for targeted ACF activities. From a programmatic perspective, however, such selection bias may be less relevant. Identified hot-spots could be marketplaces or clinics near “true” TB hot-spots and this may be sufficiently useful information for program teams since the objective is for ACF interventions to consistently provide higher yields. A very limited number of camps were conducted in military cantonments that include several high-income residential areas. These areas were therefore not adequately assessed for spatial variation in TB risk.

Challenges in participant recruitment included lower representation of females. Due to cultural reasons, women may have been hesitant to take part in screening camps in public locations and this biases the data towards males^32,33^. Since children aged less than 12 years were generally not screened, the study population was older than the general population in the country. The elderly population and those with disabilities may have also not taken part in screening. It is possible that such people with TB were residing near camp sites and were missed, affecting the number of hotspots identified. These constraints were however, applicable to all camps and would therefore have limited the bias towards individual clusters. Sputum expectoration and quality also proved challenging in the field and this may have also reduced the number of hotspots identified in the MTB positivity analysis. Limited sputum quantity, salivary samples, food particles and betel nut contaminants were frequent problems identified at the laboratory and these have also been described by other ACF programs and diagnostic facilities^34,35^. People with abnormal X-rays were encouraged to visit the nearest SZ centers to deposit morning samples and provided phone-call reminders to increase testing rates. Additional limitations include the sensitivity of the CAD software for screening for TB and of Xpert as a diagnostic test, particularly for pauci-bacillary disease^36^. It is likely that a number of individuals that were started treatment on clinical basis may have early-stage disease and would have converted to bacteriological positivity in the future. This may have accounted for the large proportion of camps with no positive results and limited the number of hotspots identified. While there was some overlap between the two outcome measures, separate clusters for abnormal CXRs and MTB+ were also identified. These may have resulted from differences in sputum-collection techniques between field-teams or could also reflect hotspots of early and subclinical TB. Future studies may consider a low cutoff for the CAD scoring, use of Xpert Ultra and novel biomarkers to improve sensitivity for early case-detection^37^.

## Methods

### Study design and setting

We analyzed retrospective data from mobile chest X-ray supported active case-finding camps conducted in Karachi, Pakistan as part of an active case-finding intervention for TB, from July 2018 to March 2020. Karachi is the most populous city of Pakistan with an estimated population of over 16 million, and a population density of over 24,000 people per square kilometer^38^. Pakistan is a relatively young country with a median age of is 22.8 years^39^. The last prevalence survey conducted in Pakistan was in 2010–11 and demonstrated increasing rates of TB with age. TB prevalence was 179.5 per 100,000 among 15–24 years, 391.9 per 100,000 among 45–54 years and per 690.5 per 100,000 among those 65 years and above^40^.

### Overview of the TB program in Pakistan

Policy-making and technical guidance for TB control in Pakistan is overseen by the National TB Program (NTP) at the federal level, whereas intervention planning, implementation and surveillance is conducted by four Provincial TB Programs (PTPs), including in Sindh province, the setting of this study. In addition to activities conducted by the public-sector, various components and interventions of the TB program are implemented by private-sector partners in collaboration with the PTPs. In 2017, there were two private-sector Principal Recipients (The Indus Hospital and Mercy Corps) and 8 implementing Sub-Recipients (SR) that received support to implement TB control activities under the Global Fund country-allocation to Pakistan. This active case-finding intervention was part of a larger private-sector TB case-detection intervention conducted by Community Health Solutions (CHS) as a sub-recipient to The Indus Hospital. The project was developed in collaboration with the Sindh PTP and received approvals from the Health Department, Government of Sindh that administers the PTP. All project activities were monitored by the PTP, cases detected were notified as part of existing TB surveillance mechanisms and TB patients were treated according to national guidelines.

### Participant enrollment and case-detection algorithm

Community screening camps were conducted using a fleet of mobile-vans with installed digital radiography equipment for chest X-rays (CXR). A total of 5 teams consisting of a screener, radiographer, mobile-van driver and two supervisors were responsible for implementing the camps. Site selection was primarily led by field-teams based on local knowledge and consultation with district health authorities. Camps were often conducted in collaboration with general physicians (GPs), pharmacies and dispensaries to improve turnout. Community mobilization was conducted through announcements in mosques and restaurants, advocacy with community influencers and distribution of communication materials (flyer and banners). Use of loudspeakers and door-to-door visits by community workers supported mobilization on the day of the camp.

Field-teams targeted a total of 150 screening per day and a high-throughput of a CXR examination every 3–4 min, over an 8 h operational period of the van. To facilitate rapid screening, limited demographic data was collected for screening and details of participant ethnicity, residential address, etc., were not captured. All individuals greater than 12 years of age who approached the mobile van during the camp were eligible for screening. All participants were offered screening using a digital CXR supported by computer-aided detection (CAD) software irrespective of symptoms^41^. Children under the age of 12 were generally not screened due to limitations of CXRs as a diagnostic tool for TB in this age-group and lack of validity of the CAD software for children at the time of the intervention. People with presumptive TB were identified based on the results of the CXR and were counselled to submit a sputum sample for Xpert MTB/RIF (Xpert) testing. In certain instances, sputum was also collected from people with normal chest X-rays, if recommended by GPs based on patient symptomatology or clinical examination. A standardized training on TB diagnosis and treatment developed by the NTP was administered to GPs prior to their enrollment within the TB screening network. These guidelines served as the basis for sputum-collection of individuals with normal CXRs^42^. Sputum induction was carried out where feasible through ultrasonic nebulization and mucolytic agents. Centralized Xpert testing was conducted in private TB diagnostic and treatment centers called *Sehatmand Zindagi (Healthy Life)* with the use of a sputum transport system*.* Samples were evaluated for sputum quantity and quality prior to Xpert testing. Samples with food-particles, insufficient sputum or saliva were rejected for testing. Individuals with MTB-positive results on Xpert were initiated on anti-TB treatment (ATT) at the centers and notified to Provincial Tuberculosis Program (PTP), Sindh. Individuals with unavailable or negative Xpert results but with high suspicion of TB after clinical evaluation by medical officers at the centers were started on ATT. All individuals were offered X-ray screening, Xpert testing and TB treatment services free of charge.

### Ethical approval and consent to participate

An ethical approval for this study was sought with the Ethics Review Committee (ERC) at KIT Royal Tropical Institute, Netherlands. The ERC provided a waiver for a full ethical review for this study as the data-analysis was retrospective and all individual-level variables were de-identified prior to sharing of the dataset with the researchers. An informed consent was verbally obtained from the participants prior to screening and they were treated according standard guidelines of the Pakistan National TB Program. The research poses no risks to participants and does not give any rise to the disclosure of the participants’ identity. All methods performed in the study were in accordance with the ethical standards of the institutional research committee and with the 1964 Helsinki Declaration and its later amendments.

### Data collection

All data utilized in this study was retrieved from internal project data of CHS that is stored on a centralized Health Management Information System (HMIS). The HMIS is hosted on a password protected cloud-server with appropriate encryptions and access is restricted to authorized staff. A unique identifier (ID) was generated for each camp on the HMIS at the time of camp-planning. A custom-built android application was installed on tablet devices with 4G connectivity and provided to community workers for data-capture. Demographic data including age, gender, contact details and test IDs were recorded. Global Positioning System (GPS) coordinates were recorded at camp sites using the tablet devices, prior to start of activities. At the conclusion of camps, data was synced with the HMIS and linked to the relevant camp IDs. In the absence of accurate home addresses, patients were assumed to be residents of the same UC where the camps were conducted.

### Statistical analysis

Frequency analysis was carried out for participant demographics and TB case-detection cascade indicators at the camp-level. Continuous variables were expressed as means (and standard deviations) or medians (and interquartile range). Camps for which no GPS coordinates were available were excluded from the analysis for this study. All data were aggregated to the chest camp level in Microsoft Excel, plotted in QGIS, in which the Zonal Statistics tool was used to summarize chest camp data to the union council level using union council polygon shape files.

Outcomes of interest for the spatial analyses were MTB positivity (MTB+) and X-ray abnormality rates, calculated as the number of bacteriologically confirmed TB cases and number of abnormal X-rays, per total number of individuals screened, respectively in each camp. Individuals enrolled for ATT based on clinical evaluation (without bacteriologically confirmed TB) were not included in the analysis. This was carried out to remove biases arising from subjective clinical decision-making between medical officers. The outcomes were analyzed for spatial heterogeneity using: (1) aggregated data at the UC-level, and (2) point-pattern analyses. Local Indicators of Spatial Autocorrelation (LISA) analyses were performed and local Moran’s I statistics were calculated to test for spatial autocorrelation for chest camp MTB+ rates and abnormal X-ray rates aggregated to the respective UC in Karachi. The LISA analysis was utilized to identify high or low clustering of MTB+ rates and abnormal X-ray rates at the UC level. The LISA analyses were performed using a first order queen contiguity (implying that only directly bordering union councils or those that share a vertex were considered as neighbors in the analysis). LISA tests were run on empirical Bayes smoothed rates of MTB positivity (MTB positive cases divided by chest camp participants) and chest X-ray abnormality (abnormal chest X-rays divided by chest camp participants), to account for varying underlying population at the UC-level.

Chest camp level MTB+ rates and abnormal X-ray rates were also analyzed for spatial autocorrelation using the Getis Ord Star (GI*) analysis. This was carried out using (1) distance-based and (2) contiguity (neighbor) based methods. In the distance-based approach, camps within a distance band of 1 km around each other were analyzed for spatial autocorrelation. In the contiguity-based approach, thiessen polygons were generated around each chest camp coordinate and analyzed for spatial autocorrelation using queen contiguity (the contiguity-based approach is presented in Supplementary Fig. 4. Point-patterns have been presented using administrative boundaries demarcating UCs instead of polygons for clarity). The GI* analysis identified point locations with spatially statistically significant high or low clustering of MTB+ and abnormal X-ray rates. Association between MTB+ and abnormal X-rays was investigated using logistic regression with MTB+ as the outcome variable and abnormal X-rays (defined as CAD4TB ≥ 50) as the explanatory variable. Frequency analysis was carried out using Stata version 13 (College Station, TX: StataCorp LP). Spatial analyses were conducted using GeoDa’s LISA and GI* tests and exported to QGIS for mapping and visualization^43^.

## Conclusion

Statistically significant spatial variation was identified in yield of bacteriologically positive TB cases and in abnormal CXRs through active case-finding in Karachi. This suggests that TB transmission in the city is clustered in certain areas that can be targeted for screening. Further research is required to investigate clustering of TB cases and its predictors in other parts of Pakistan. Prospective studies investigating yields and comparative cost-effectiveness of targeted screening in areas predicted to be higher risk should also be conducted in Pakistan and other high TB burden countries.

## Supplementary Information

Supplementary Information.

## Data Availability

The dataset used for analysis during the current study is available from the corresponding author on reasonable request.
