# Supplementary material for: A spatial analysis of TB cases and abnormal X-rays detected through active case-finding in Karachi, Pakistan
Source: Sci Rep. 2023 Jan 24;13:1336. doi: 10.1038/s41598-023-28529-9 (PMC9873642; doi:10.1038/s41598-023-28529-9)
Supplement: Supplementary file 1 — Supplementary Information. [file 41598_2023_28529_MOESM1_ESM.docx]

**A spatial analysis of TB cases and abnormal X-rays detected through active case-finding in Karachi, Pakistan**

**Syed Mohammad Asad Zaidi ^1^, Wafa Zehra Jamal * ^1^, Christina Mergenthaler ^2^, Kiran Sohail Azeemi ^1^, Nick Van Den Berge ^2^, Jacob Creswell ^3^, Aamir Khan ^4^, Saira Khowaja ^5^, Shifa Salman Habib^1^**

^1^ Community Health Solutions, Pakistan

^2^ KIT Royal Tropical Institute, Netherland

^3^ The Stop TB Partnership, Geneva, Switzerland

^4^ IRD Global, Singapore

^5^ Indus Health Network, Karachi, Pakistan

*** (corresponding author)**

[wafa.zj@](mailto:wafa.zj@)yahoo.com

00923002889961

ORCID:0000-0002-3509-5260


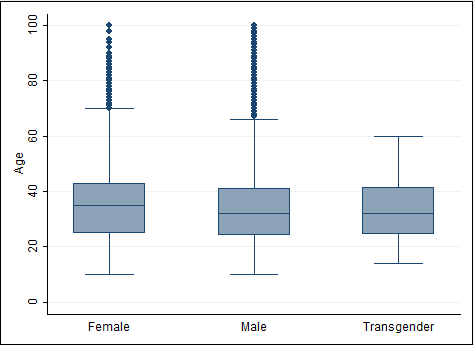
**Supplementary Figure 1:** Participant demographics during mobile chest X-ray supported active case-finding camps in Karachi, Pakistan (July 2018- March 2020).

**Supplementary Figure 2:** Crude MTB+ rates of mobile chest X-ray supported active case-finding camps in Karachi, Pakistan (July 2018- March 2020). QGIS Geographic Information System v3.26.3. QGIS.org, 2022. QGIS Association. [http://www.qgis.org](http://www.qgis.org/).


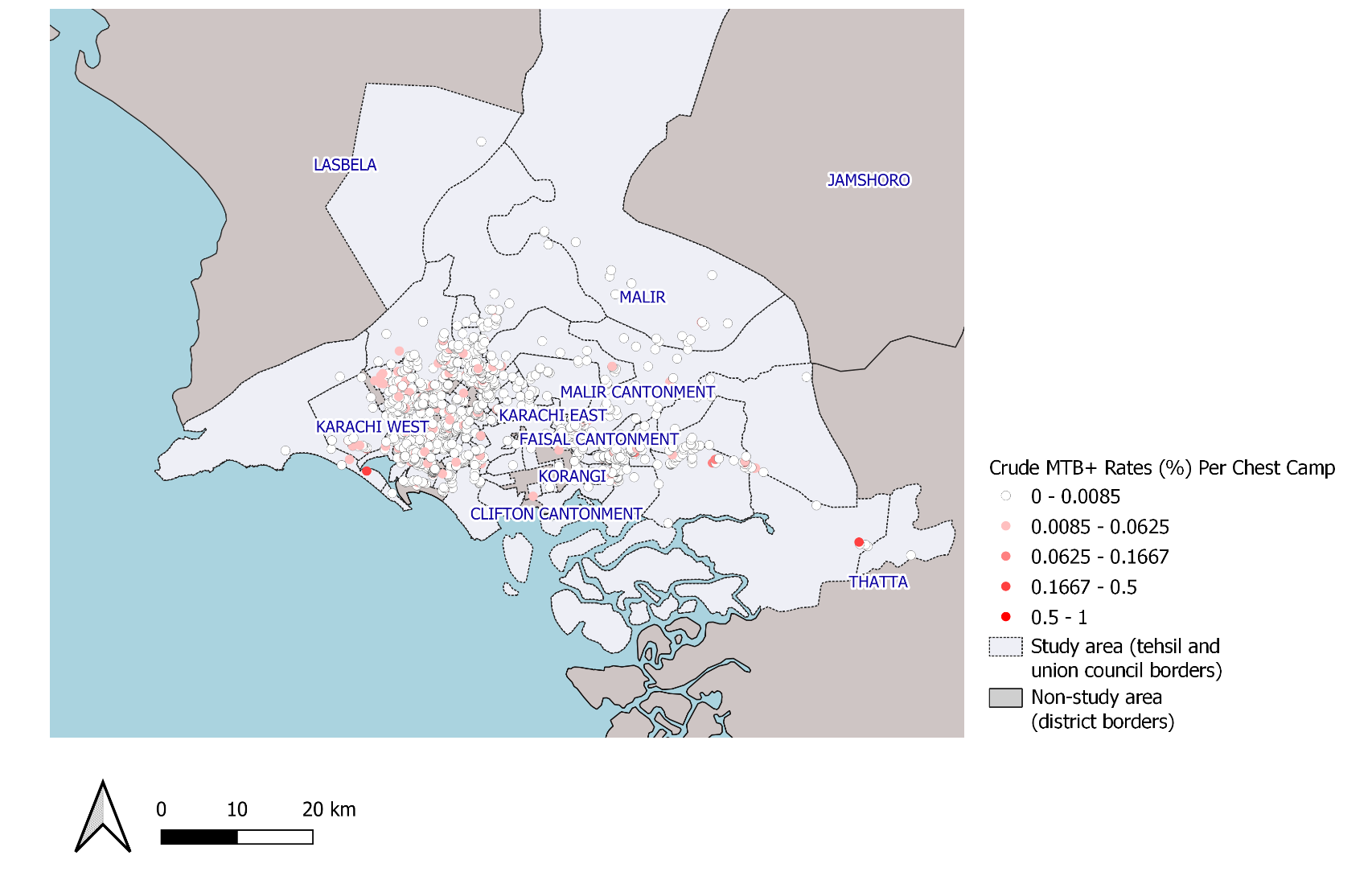


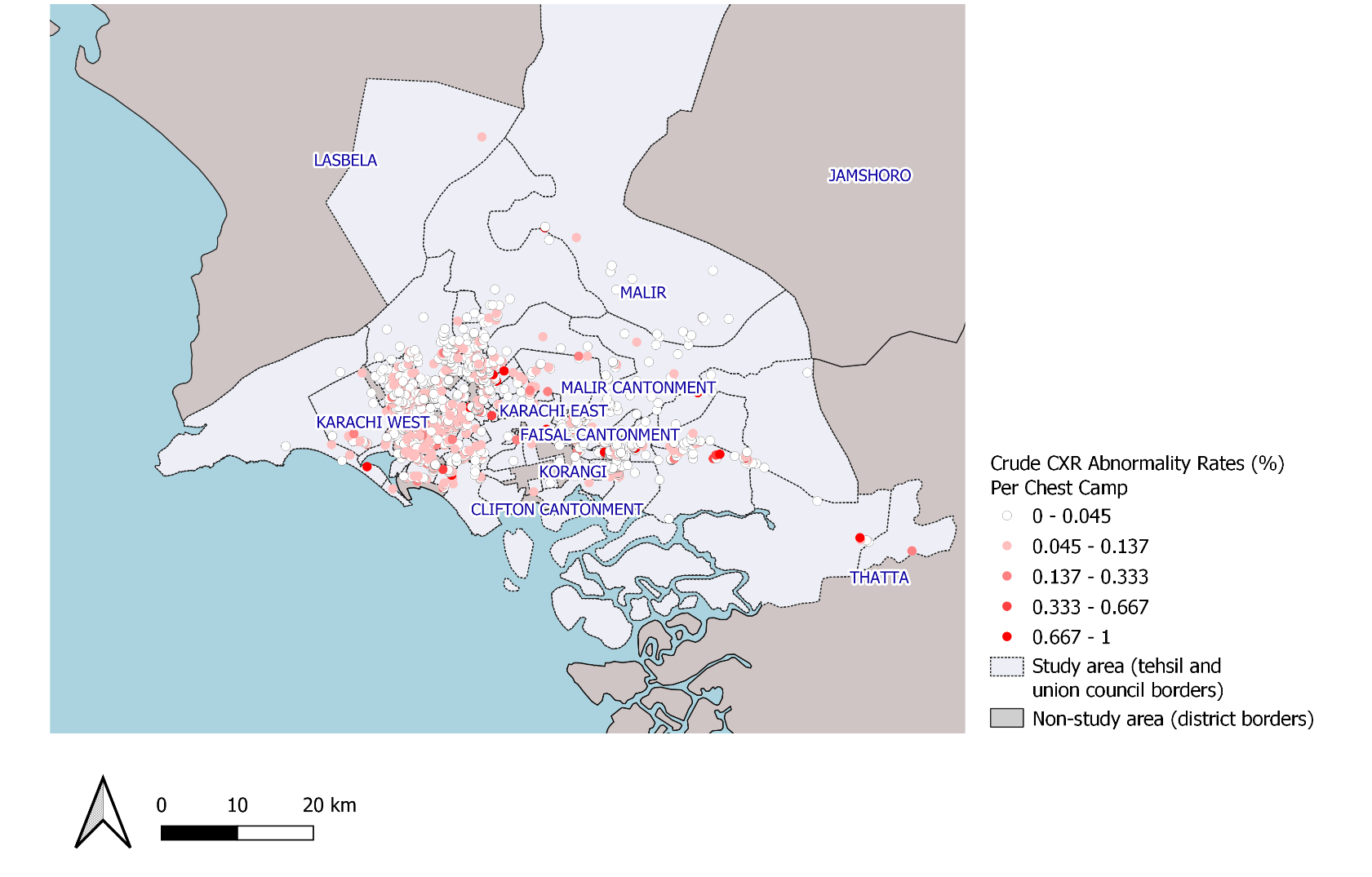
**Supplementary Figure 3:** Crude abnormal X-ray rates of mobile chest X-ray supported active case-finding camps in Karachi, Pakistan (July 2018- March 2020). QGIS Geographic Information System v3.26.3. QGIS.org, 2022. QGIS Association. [http://www.qgis.org](http://www.qgis.org/).

**Supplementary Figure 4 a**: GI* Analysis (contiguity-based method) for MTB positivity ratios from locations of mobile chest X-ray supported active case-finding camps in Karachi, Pakistan (July 2018- March 2020). In the contiguity-based approach a Thiessen polygon was generated around each point and neighboring point polygons were evaluated for high or low MTB positivity. For clarity and consistency, the polygons are not shown and the point-patterns are overlaid on administrative boundaries demarcating union-councils. A High-high result indicates clustering of GPS locations of camps with high MTB positivity. A Low-low result indicates clustering of GPS locations of camps with low MTB positivity. QGIS Geographic Information System v3.26.3. QGIS.org, 2022. QGIS Association. [http://www.qgis.org](http://www.qgis.org/).

**
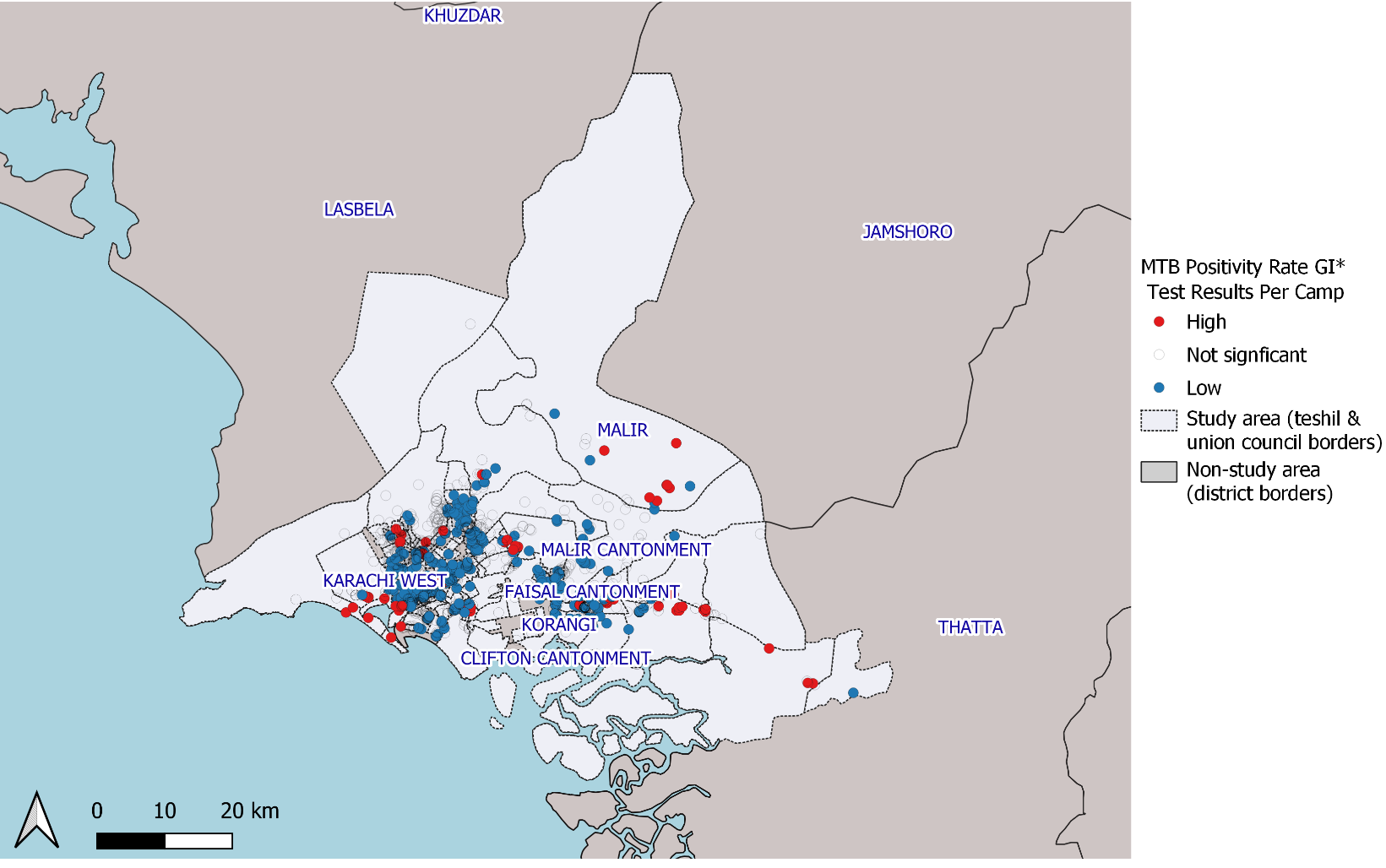
**

**Supplementary Figure 4**b: GI* Analysis (contiguity-based method) for abnormal X-rays ratios from locations of mobile chest X-ray supported active case-finding camps in Karachi, Pakistan (July 2018- March 2020). In the contiguity-based approach a Thiessen polygon was generated around each point and neighboring point polygons were evaluated for high or low abnormal X-ray ratios. For clarity and consistency, the polygons are not shown and the point-patterns are overlaid on administrative boundaries demarcating union-councils. A High-high result indicates clustering of GPS locations of camps with high abnormal X-ray ratios. A Low-low result indicates clustering of GPS locations of camps with low abnormal X-ray ratios. QGIS Geographic Information System v3.26.3. QGIS.org, 2022. QGIS Association. [http://www.qgis.org](http://www.qgis.org/).


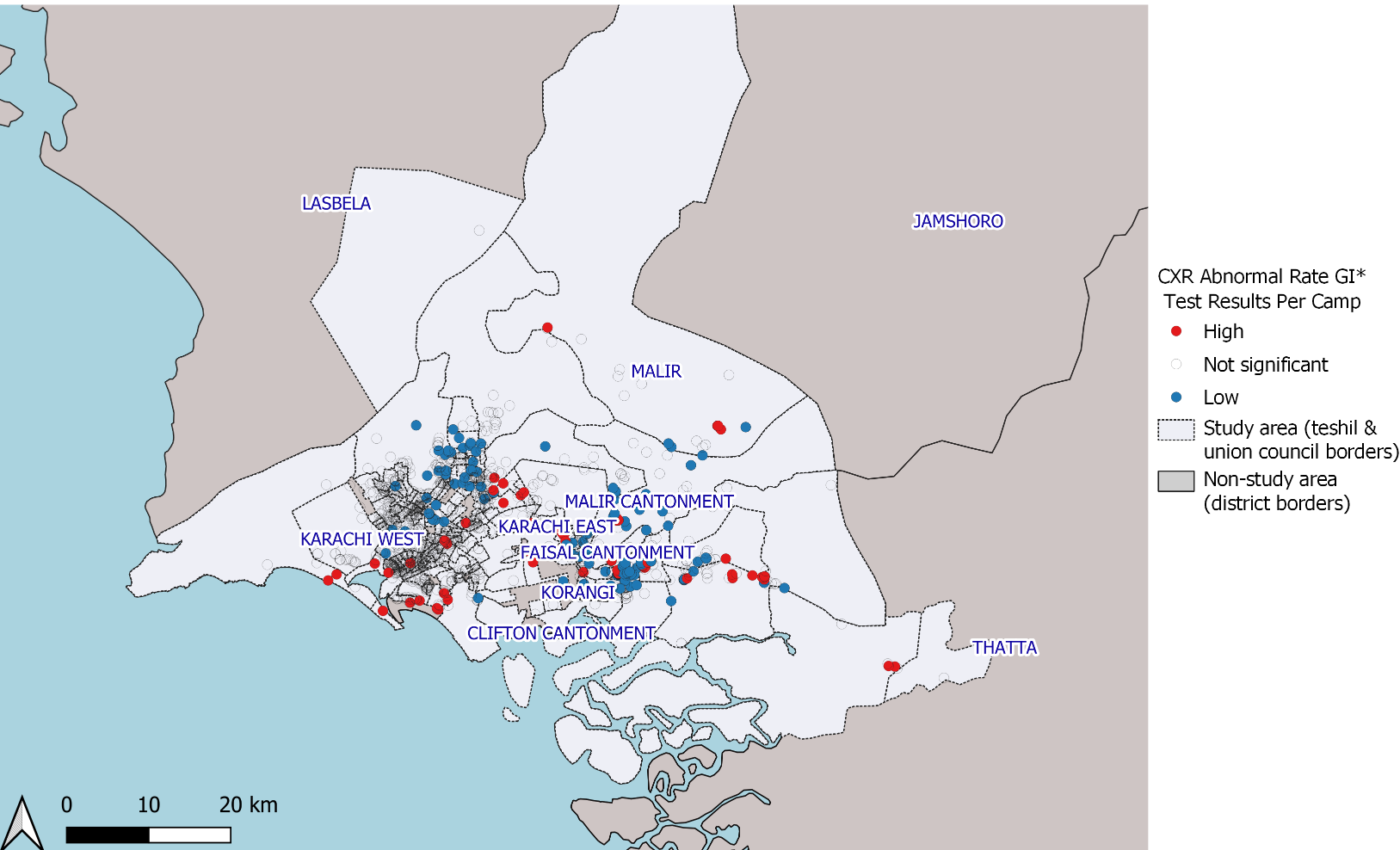


**Supplementary Figure 5**: Karachi’s socioeconomic divide is illustrated in the sharpest context from a mountain ridge separating Orangi, an industrial area and with vast, crowded slum dwellings in the west, from North Nazimabad Town, a planned upper-middle class neighborhood on the east. Active case-finding with GPS mapping of camp locations identified a number of TB hotspots towards the west whereas cold-spots were identified east, highlighting the need for neighborhood-level analyses for TB elimination in mega-cities.

Image source: Google Earth version 9.171.0.0. (2022). Karachi, Pakistan. Maxar Technologies.

**
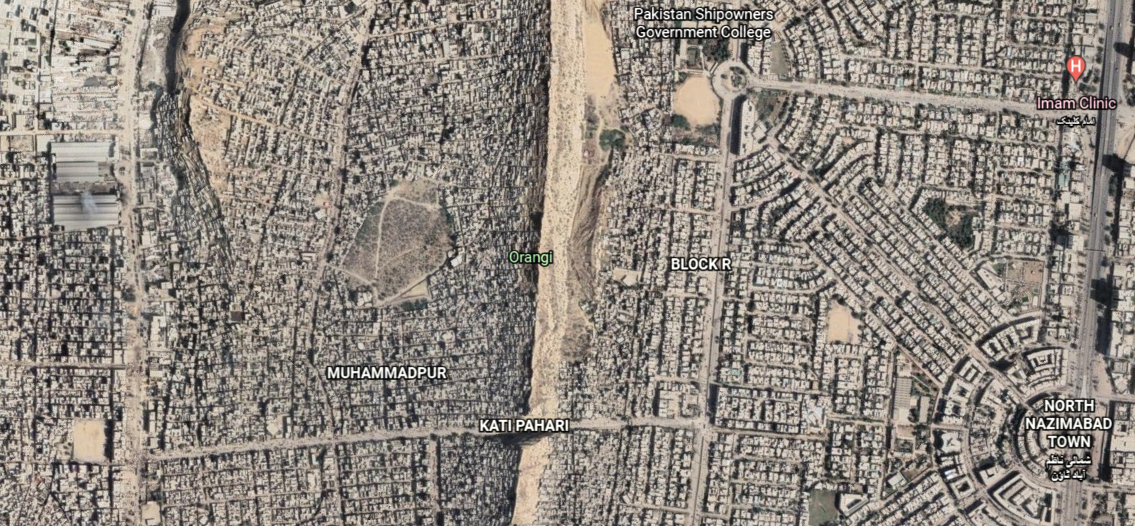
**<https://earth.google.com/web/@24.94515183,67.03422279,58.24267172a,2461.70673456d,35y,31.5244826h,0t,0r>

**Supplementary Table 1: High-high MTB positivity locations identified from mobile chest X-ray supported active case-finding camps in Karachi, Pakistan (July 2018- March 2020).**

| **Longitude** | **Latitude** | **Area Name** | **Also identified High-high abnormal X-ray cluster** |
| --- | --- | --- | --- |
| 67.389 | 25.0957 | Lal Bux Kachelo Goth, on Langho Nadi, a Malir River tributary, off the Karachi-Hyd Motorway | Yes |
| 67.2826 | 25.0848 | Radho Jokhio Goth, near Konkar |  |
| 67.375 | 25.0337 | Pehlwan Jokhio Goth, off Karachi Hyderabad Motorway, on Malir River tributary | Yes |
| 67.3758 | 25.0337 | Kathore, off Karachi Hyderabad Motorway, on Malir River tributary | Yes |
| 67.3792 | 25.0293 | Safar Khoso Goth, Kathore, off Karachi Hyderabad Motorway, on Malir River tributary | Yes |
| 67.3495 | 25.0153 | Community Camp at Kathore, behind Al Jeddah Restaurant, Karachi Hyderabad Motorway |  |
| 67.3606 | 25.0104 | Next to Al-Jeddah Restaurant, on Karachi Hyderabad Motorway |  |
| 67.5913 | 24.7402 | Gharo, near PSO Pump, off the National Highway 5 | Yes |
| 67.5837 | 24.7409 | Gharo, near Malook Hotel, off the National Highway 5 |  |
| 67.5265 | 24.792 | Dhabeji, Indus Jute Mill, behind Habib Bank |  |
| 67.3852 | 24.8717 | Jiyal Shah Jillani Goth, off National Highway 5, near Eastern Bypass | Yes |
| 67.3724 | 24.8706 | Sehatmand Zindagi Center, Gulshan-e-Hadeed, near Al-Khidmat Fareeda Yaqoob Hospital |  |
| 67.36293 | 24.85456 | Gohram Bugti Goth, near Gulshan-e-Hadeed |  |
| 67.39802 | 24.85373 | Pipri, Bin Qasim Town |  |
| 67.39289 | 24.85262 | Pipri, Bin Qasim Town | Yes |
| 67.38939 | 24.84802 | Pipri, Bin Qasim Town |  |
| 67.39357 | 24.84809 | Pipri, Bin Qasim Town | Yes |
| 67.4324 | 24.8501 | Ghaghar Phatak, Bin Qasim Town | Yes |
| 67.4326 | 24.8498 | Ghaghar Phatak, Bin Qasim Town | Yes |
| 67.4321 | 24.8464 | Ghaghar Phatak, Bin Qasim Town | Yes |
| 67.2463 | 24.8562 | Hasan Panhwar Goth, opposite Sindhi Jamat Cooperative Housing Society, near District Jail Malir |  |
| 67.2965 | 24.8647 | Razzaqabad, near Qasim Port Road |  |
| 67.2901 | 24.8671 | Razzaqbad, Police Training Center |  |
| 67.2839 | 24.8633 | Abdullah Goth, near Shah Latif Town |  |
| 67.284 | 24.8631 | Abdullah Goth, near Shah Latif Town | Yes |
| 67.2861 | 24.8608 | Dur M Khurdoos Goth, near Shah Latif Town | Yes |
| 67.2867 | 24.8589 | Dur M Khurdoos Goth, near Shah Latif Town | Yes |
| 67.1016 | 25.0493 | Lyari Basti, Sector 36, Surjani Town |  |
| 67.09191 | 24.94709 | Bakhar Goth, Sohrab Goth, behind Chapal Gardens |  |
| 67.1353 | 24.9491 | Sachal Goth, behind Karachi University | Yes |
| 67.1381 | 24.9518 | Sachal Goth, behind Karachi University | Yes |
| 67.139 | 24.9525 | Sachal Goth, behind Karachi University | Yes |
| 67.1479 | 24.93793 | University Road, near Old Ravians Housing Society |  |
| 67.1549 | 24.9423 | Usmani Town near Sachal Police Station |  |
| 67.15066 | 24.94324 | Usmani Town near Gabol Goth Road |  |
| 67.1508 | 24.94337 | Usmani Town near Gabol Goth Road |  |
| 67.1549 | 24.9423 | Usmani Town near Sachal Police Station |  |
| 67.023 | 24.9509 | Baloch Goth, near Qasbaa Colony |  |
| 67.0185 | 24.9491 | Baloch Colony, near Qasba Colony |  |
| 66.9824 | 24.9601 | Gulzar-e-Madina Colony, near Chishti Nagar |  |
| 66.9719 | 24.9591 | Muhammad Khan Colony, near Chishti Nagar |  |
| 66.9807 | 24.9497 | Tauheed Colony, Orangi Town |  |
| 66.98 | 24.9482 | Tauheed Colony, Orangi Town |  |
| 67.083 | 24.8579 | KAECHS, near Allied Bank, Mehmoodabad |  |
| 67.078 | 24.8537 | Near Mehmoodabad Graveyard, Mehmoodabad |  |
| 67.0853 | 24.8525 | Manzoor Colony, near Muhammadi Mosque |  |
| 67.07537 | 24.84767 | Azam Basti, near Faizan e Jamal Mustafa Mosque |  |
| 67.0785 | 24.847 | Azam Basti, near Madina Masjid |  |
| 67.0845 | 24.84696 | Manzoor Colony, Sector E |  |
| 67.0404 | 24.8329 | Dehli Colony near Askari 1 |  |
| 67.042 | 24.8938 | 3 Hatti, Liaqatabad |  |
| 67.0422 | 24.8938 | 3 Hatti, Liaqatabad |  |
| 67.0442 | 24.892 | 3 Hatti, Liaqatabad | Yes |
| 67.0382 | 24.8904 | Martin Quarters West, opposite Rehmani Gardens |  |
| 67.0394 | 24.8883 | Martin Quarters West, near Bijli Ground |  |
| 67.0454 | 24.8899 | Martin Quarters East, near Jamia Baghdadi Mosque | Yes |
| 67.0454 | 24.8898 | Martin Quarters East, near Jamia Baghdadi Mosque | Yes |
| 66.9004 | 24.8452 | Hawkes Bay, near Kakapir Road and Hawkes Bay Drive intersection | Yes |
| 66.9107 | 24.8526 | Younusabad, Hawkes Bay | Yes |
| 66.9672 | 24.8081 | Salehabad, Manora | Yes |
| 66.9817 | 24.8246 | Kemari, near Ziauddin Hospital |  |
| 66.9787 | 24.8481 | Machar Colony |  |
| 66.974 | 24.8546 | Machar Colony |  |
| 66.9573 | 24.8657 | Machar Colony | Yes |
| 66.9333 | 24.8668 | Grax Colony, Maripur |  |
| 66.9316 | 24.8682 | Grax Colony, Maripur |  |
| 66.9289 | 24.8692 | Grax Colony, Maripur, near St Mathew's Catholic Church |  |

**Supplementary Table 2: High-high abnormal chest X-ray locations identified from mobile chest X-ray supported active case-finding camps in Karachi, Pakistan (July 2018- March 2020).**

| **Longitude** | **Latitude** | **Area Name** |
| --- | --- | --- |
| 67.5913 | 24.7402 | Gharo, off the National Highway 5 |
| 67.375 | 25.0337 | Pehlwan Jokhio Goth, off Karachi Hyderabad Motorway, on Malir River tributary |
| 67.3758 | 25.0337 | Kathore, off Karachi Hyderabad Motorway, on Malir River tributary |
| 67.3792 | 25.0293 | Safar Khoso Goth, Kathore, off Karachi Hyderabad Motorway, on Malir River tributary |
| 67.1679 | 25.1535 | Goth Gohram, Kirthar Park Road, Gadap |
| 67.389 | 25.0957 | Lal Bux Kachelo Goth, on Langho Nadi, a Malir River tributary, off the Karachi-Hyd Motorway |
| 67.3386 | 24.9479 | Darsano Channo, near Cadet College Gadap |
| 67.338 | 24.8477 | Sindhi Goth near Bin Qasim Railway Station |
| 67.25422 | 24.91844 | Sehatmand Zindagi Center, Memon Goth, near Habib Bank Limited |
| 67.287 | 24.8618 | Dur M Khuroos Goth, near Shah Latif Town |
| 67.2541 | 24.9185 | Memon Goth, |
| 67.2529 | 24.8577 | Yousuf Goth, opposite District Jail Malir |
| 67.2449 | 24.8547 | Hasan Panhwar Goth, opposite Sindhi Jamat Cooperative Housing Society |
| 67.2101 | 24.857 | National Highway 5, Quiadabad, near Pakistan Swedish Institute of Technology |
| 67.2458 | 24.8691 | Saleh Muhammad Goth, Bin Qasim Town |
| 67.103 | 24.9703 | Quetta Town, Block 3 |
| 67.1114 | 24.9759 | Sector 4A, Dehli Mercantile Society Scheme 33 |
| 67.1019 | 24.9551 | Gulzar-e-Hijri Scheme 33, Sector 15-A |
| 67.1138 | 24.9635 | Suparco Road, near Corniche CHS |
| 67.1353 | 24.9491 | Sachal Goth, behind Karachi University |
| 67.139 | 24.9525 | Sachal Goth, behind Karachi University |
| 67.14164 | 24.90983 | Pehelwan Goth |
| 67.14912 | 24.91505 | Bhittaiabad, near Muhammadi Mosque |
| 67.1467 | 24.8797 | Shah Faisal Colony, Block 2, behind Nisar Hussain Shaheed Park |
| 67.1787 | 24.8779 | Alfalah Housing Society, near Ameer-e-Muaviya Mosque |
| 67.1804 | 24.882 | Siddique Goth, near Malir Halt |
| 67.1898 | 24.8929 | Saudabad, C Area, opposite Liaquat Government College for Girls |
| 67.1837 | 24.9082 | Model Colony Sheet 27 Extension |
| 67.1855 | 24.903 | Model Colony, Liaqat Ali Khan Road, near Jamia Moque Madina |
| 67.18874 | 24.90243 | Model Colony, Liaqat Ali Road, near DHO Malir, Model Town Health Office |
| 67.1882 | 24.9013 | Model Colony, near KICA, Karachi Institute of Cullinary Arts |
| 67.1871 | 24.9008 | Model Colony, Hashim Raza Road |
| 67.3852 | 24.8717 | Jiyal Shah Jillani Goth, off National Highway 5, near Eastern Bypass |
| 67.39289 | 24.85262 | Pipri, Bin Qasim Town |
| 67.39357 | 24.84809 | Pipri, Bin Qasim Town |
| 67.4324 | 24.8501 | Ghaghar Phatak, Bin Qasim Town |
| 67.4326 | 24.8498 | Ghaghar Phatak, Bin Qasim Town |
| 67.4321 | 24.8464 | Ghaghar Phatak, Bin Qasim Town |
| 67.284 | 24.8631 | Abdullah Goth, near Shah Latif Town |
| 67.2861 | 24.8608 | Dur M Khurdoos Goth, near Shah Latif Town |
| 67.2867 | 24.8589 | Dur M Khurdoos Goth, near Shah Latif Town |
| 67.1381 | 24.9518 | Sachal Goth, behind Karachi University |
| 67.0454 | 24.8898 | Martin Quarters East, near Jamia Baghdadi Mosque |
| 67.0442 | 24.892 | 3 Hatti, Liaqatabad |
| 66.9672 | 24.8081 | Salehabad, Manora |
| 66.9573 | 24.8657 | Machar Colony |
| 66.9107 | 24.8526 | Younusabad, Hawkes Bay |
| 66.9004 | 24.8452 | Hawkes Bay, near Kakapir Road and Hawkes Bay Drive intersection |
| 67.0003 | 24.8181 | Sehatmand Zindagi Center, Shireen Jinnah, on Shahrah-e-Ghalib |
| 67.0119 | 24.8206 | Gulshan-e-Sikandarabad, Block 5, Boat Basin |
| 67.0417 | 24.8296 | Dehli Colony near Askari 1 |
| 67.0347 | 24.8098 | Shah Rasool Colony, Clifton |
| 67.033 | 24.8114 | Shah Rasool Colony, Clifton |
| 67.0454 | 24.8899 | Martin Quarters East, near Jamia Baghdadi Mosque |
| 66.8263 | 24.8644 | Ramzan Goth, Hawkes Bay, Maripur Road |
